# Dig deeper when it does not make sense: Juvenile xanthomas due to sitosterolemia

**DOI:** 10.1002/jmd2.12161

**Published:** 2020-08-20

**Authors:** Sharmila Kiss, Joy Yaplito Lee, James Pitt, Duncan MacGregor, Jane Wallace, Melanie Marty, Natasha J. Brown

**Affiliations:** ^1^ Department of Metabolic Medicine The Royal Children's Hospital Parkville Victoria Australia; ^2^ Victorian Clinical Genetics Services Murdoch Children's Research Institute Parkville Victoria Australia; ^3^ Department of Paediatrics University of Melbourne Melbourne Victoria Australia; ^4^ Department of Anatomical Pathology The Royal Children's Hospital Parkville Victoria Australia

**Keywords:** juvenile xanthomas, sitosterolemia

## Abstract

Sitosterolemia is an extremely rare autosomal recessive disease caused by mutations in either *ABCG5* or *ABCG8*, which encode for a sterol efflux transporter (sterolin) that pumps sterols out into the intestinal lumen or into bile. This leads to progressive accumulation of plant sterols in blood and tissues. Clinical presentation is variable and may include xanthoma, arthritis, thyroid dysfunction, premature atherosclerotic disease, splenomegaly, and hematologic manifestations. We report a child presented with multiple xanthomas at age 5.5 years, located on the elbow, knee, and toe. Juvenile xanthogranuloma was considered based on histopathologic findings. At 8 years of age, a lipid profile showed markedly elevated total cholesterol (9.4 mmol/L) and low‐density lipoprotein cholesterol (LDL‐C, 7.4 mmol/L). Simvastatin therapy was initiated, however, the lipid profile was persistently abnormal. At age 8.5 years, genetic testing identified two novel variants: (NM_022437.3[ABCG8]:c.1444del;p.Leu482Trpfs*40) and (NM_022437.3[ABCG8]:c.1640T>C;p.Leu547Pro) in the *ABCG8* gene. Plasma sitosterol was subsequently found to be very high, confirming the diagnosis. She was started on a low plant sterol and cholesterol diet for 6 weeks with insignificant response and therefore ezetimibe (10 mg daily) was added. This resulted in significant reduction of cholesterol, LDL, sitosterol levels, and no further increase in the size of the xanthomas. This case emphasizes the diagnostic odyssey, the benefits of genomic testing and importance of a correct diagnosis in order to initiate appropriate therapy. It also illustrates the importance of considering rare conditions, such as sitosterolemia, as a differential diagnosis in patients with hypercholesterolemia and increased LDL‐C.

## 1 INTRODUCTION

Sitosterolemia is an extremely rare, autosomal recessive disease characterized by accumulation of plant sterols in blood and tissues.^1^ It is caused by homozygous or compound heterozygous variants in either *ABCG5* or *ABCG8*, which encode the subunits of sterolin, the sterol efflux transporter that pumps sterols out to the intestinal lumen or into bile.^2^, ^3^ Loss of function of this transporter leads to increased intestinal absorption and decreased biliary excretion of all dietary sterols and thus to progressive accumulation of sterols.^4^

There is clinical variability in the presentation of individuals with sitosterolemia, from asymptomatic to early lethality.^5^ Clinical features may include xanthoma, arthritis, thyroid dysfunction, premature atherosclerotic disease, splenomegaly, and hematologic manifestations including unexplained hemolytic anemia, macrothrombocytopenia, and abnormal bleeding.^6^, ^7^

The true prevalence of sitosterolemia is unknown. This is likely due to various factors, such as poor recognition, or misdiagnosis as familial hypercholesterolemia without an accurate plasma sterol analysis, and limited access to molecular genetic testing. Routine laboratory methods, such as lipid profile studies for determining total cholesterol levels do not distinguish plant sterols from cholesterol. Alternative methods, such as gas chromatography‐mass spectrometry (GC‐MS), are required to distinguish between cholesterol and other sterols and thus facilitate recognition of sitosterolemia.^8^, ^9^ Higher total cholesterol and phytosterol levels have been reported in pediatric as compared to adult cases; it has been postulated that the immature intestine might absorb higher amounts of cholesterol and phytosterols than that of adults.^10^

Correct diagnosis is important in order to instigate appropriate therapy, such as cholesterol and plant sterol restriction and commencement of a cholesterol absorption inhibitor. While measurement of increased plasma phytosterols can lead to the diagnosis of sitosterolemia, this testing may not be available or routinely requested during the initial diagnostic work up. In some laboratories, full sterol analysis is only initiated if less common conditions are considered in the differential diagnosis. Specific molecular diagnosis can lead to improved clinical outcomes, such as reduction in advanced atherosclerotic cardiovascular disease, and also allows appropriate genetic counseling for families regarding relatives potentially at risk, including recurrence risk for parents of an affected child.^8^, ^11^

## 2 CASE REPORT

**FIGURE 1:**
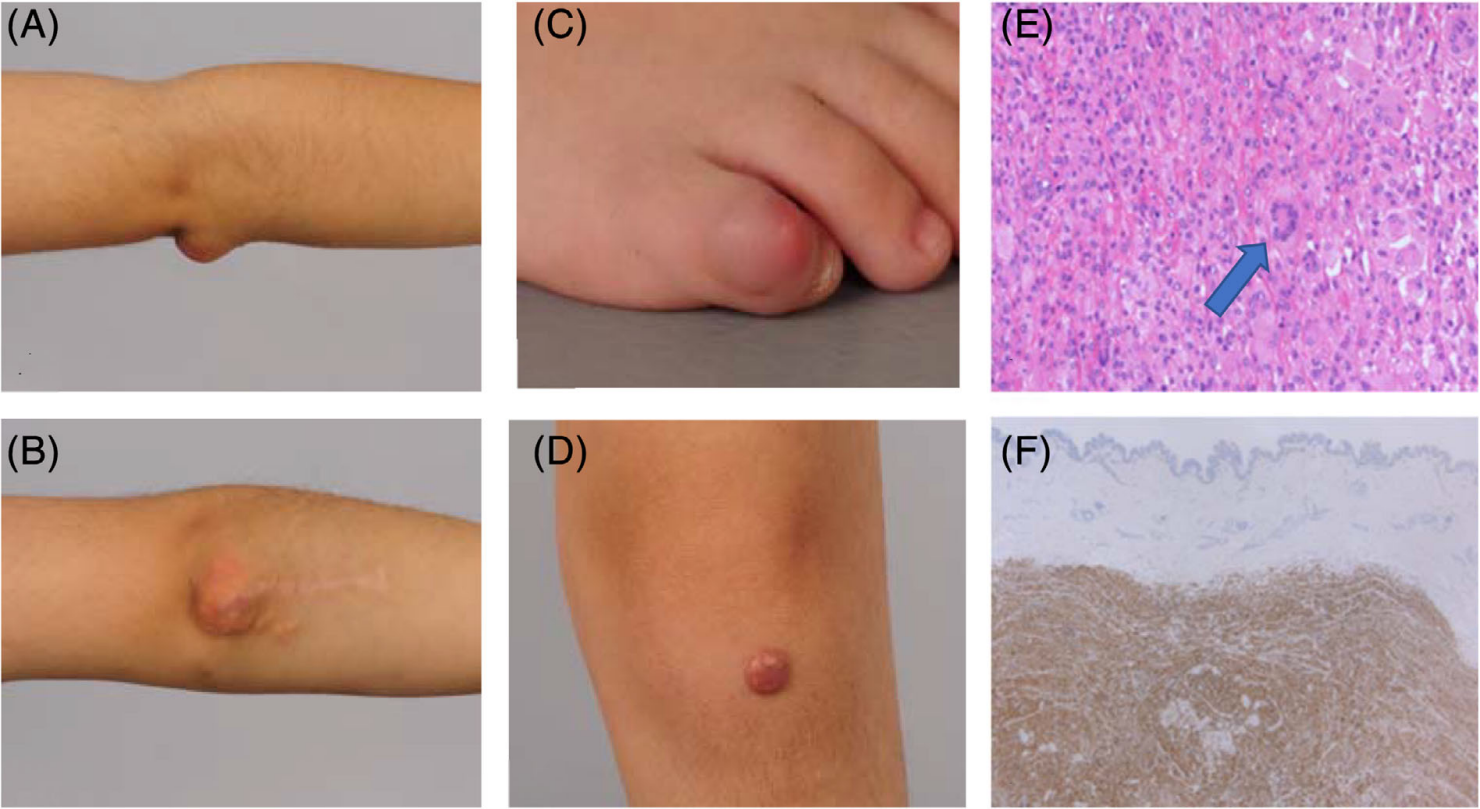
Clinical images and histopathology of skin lesions

The patient is an 8‐year‐old girl who presented with a 2.5‐year history of multiple xanthomas. She was born at 31 + 5 weeks gestation via normal vaginal delivery. Her developmental progress was age‐appropriate. Xanthomas were first noted on her right elbow (Figure 1A,B) at about 5.5 years of age. She was referred to the Plastic Surgery team by her local family doctor. The xanthomas were considered subcutaneous swellings of uncertain nature and one of them was excised for diagnostic purposes. Histopathology of the lesions showed sheets of foamy histiocytes (CD68+ CD1a− ALK−) with focally prominent Touton‐type multinucleate giant cells (Figure 1E,F). A diagnosis of juvenile xanthogranuloma was made based on histology and clinical presentation. The distribution of these lesions (legs, arms, toe) was typical for a lipid disorder. The previously excised lesion on her right elbow recurred a year later and she developed other lesions on the left knee (Figure 1C) and third lesion on the right fifth toe (Figure 1D). She was referred to the Oncology team for further opinion after recurrence of the lesions. The Oncology team saw her at 6 years of age and ordered magnetic resonance imaging (MRI) of the right elbow, which showed a subcutaneous soft tissue mass measuring 10 mm by 48 mm by 54 mm with well‐defined homogenous contrast enhancement over the olecranon. Whole‐body MRI done at 7 years of age showed a lesion with well‐defined margins measuring 6.7 mm × 6.5 mm in the anteroinferior region of the left knee. A second lobulated well‐defined lesion (22 mm × 10 mm × 17 mm) in the medial aspect of the popliteal fossa was also found. There was no obvious intracranial abnormality and there were no other focal lesions demonstrated within the abdominal or thoracic cavities.

Serum lipid profile was first performed at 8 years of age and results showed total cholesterol: 12.8 (normal range 2.5‐4.9 mmol/L), triglyceride: 1.3 (normal range 0.9‐2.0 mmol/L), high‐density lipoprotein (HDL): 1.9 (normal range 1.0‐3.0 mmol/L), and low‐density lipoprotein (LDL): 10.3 (normal range < 3.5 mmol/L). Subsequently, a history of hypercholesterolemia in both parents was documented, but no family history of coronary artery disease was reported. Mother's lipid profile: total cholesterol: 5.4 (normal range < 4.0 mmol/L), triglyceride: 0.8 (normal range < 2.0 mmol/L), HDL: 1.1 (normal range > 1.0 mmol/L), and LDL: 3.9 (normal range < 2.0 mmol/L); and father's lipid profile: total cholesterol: 7.2 (normal range < 4.0 mmol/L), triglyceride: 3.3 (normal range < 2.0 mmol/L), HDL: 1.2 (normal range > 1.0 mmol/L), and LDL: 4.5 (normal range < 2.0 mmol/L). The patient was referred to the Endocrinology team due to the abnormal lipid profile and the lesions were at this stage considered to be consistent with xanthomatosis rather than xanthogranuloma.

Simvastatin was started at 10 mg daily for 2 months and increased to 20 mg daily for the next 2 months. Repeat serum lipid profile after 3 months of treatment showed minimal response (Table 1). Liver enzymes were normal. She was also commenced on a diet low in cholesterol. Simvastatin was further increased to 40 mg daily after 4 months.

**TABLE 1:** Results of biochemical markers prior to and following treatments

	1	2	3	4	5	6	7	8	9
Total cholesterol (mmol/L)	12.8	12.7	10.1	10.7	9.4	8.9	4.9	2.8	6.1
Low‐density lipoprotein cholesterol (mmol/L)	10.3	10.3	8.0	8.6	7.4	6.8	2.8	6.0	3.9
Sitosterol (μmol/L)	‐	‐	‐	‐	476	736	310	199	145

*Note*: In this table, total cholesterol values do not include sitosterol levels, but are inclusive of other phytosterols, which are not routinely determined by our local assays. Column 1: Initial lipid profile prior to any treatment; Column 2: lipid profile on day of commencement of simvastatin 10 mg daily; Column 3: lipid profile after 2 months of simvastatin 10 mg daily; Column 4: lipid profile 2 months of simvastatin 20 mg daily; Column 5: lipid profile at the time of genetic test results, after 2 months of simvastatin 40 mg daily, prior to initiating treatment for sitosterolaemia; Column 6: lipid profile 6 weeks after diagnosis (6 weeks of dietary therapy); Column 7: lipid profile 2 months after diagnosis (8 weeks diet + 2 weeks ezetimide); Column 8: lipid profile 4 months after diagnosis (16 weeks diet + 10 weeks ezetimide); Column 9: lipid profile 6 months after diagnosis (24 weeks diet + 18 weeks ezetimide).

The patient was referred to Clinical Genetics at the age of 8 years for consideration of genetic testing of familial hypercholesterolemia. The rationale for this was to see if the patient would qualify for government‐funded access to a PCSK9 inhibitor, with a presumptive diagnosis of familial hypercholesterolemia. Limited whole exome sequencing was arranged. This involved developing a virtual gene panel including the following candidate genes: *LDLR*, *APOB*, *PCSK9*, *LDLRAP1*, *LIPA*, *ABCG5*, *ABCG8*, *SLC25A13*, *CYP27A1*, *APOE*, *LPL*. A number of differential diagnoses were considered. These included familial hypercholesterolemia and sitosterolemia.

Exome sequencing identified two variants in the *ABCG8* gene. The first was a novel frameshift variant (NM_022437.3[ABCG8]:c.1444del;p.Leu482Trpfs*40), located in exon 10 of 13. This variant is predicted to cause a frameshift at position 482 with introduction of a stop codon 40 residues downstream. This variant was classified as pathogenic. The second was a novel missense variant of uncertain clinical significance, (NM_022437.3[ABCG8]:c.1640T>C;p.Leu547Pro) in exon 11 of 13. The missense variant was absent from population databases and disease cohorts; in silico predictions are consistently pathogenic and it is located in the ABC2 membrane functional domain. Both missense and loss of function variants in this gene have been reported in association with sitosterolemia.^2^

Testing of parents subsequently showed that the mother was a carrier of the frameshift variant and the father carried the missense variant, thus confirming the variants were in trans in the proband. GC‐MS analysis of plasma showed grossly increased sitosterol 736 (<20 μmol/L), confirming the diagnosis of sitosterolemia. These biochemical results, together with the clinical presentation and parental segregation, allowed the second variant to be reclassified by the laboratory as likely pathogenic.

Subsequently, she was referred to the Metabolic Team at 9 years of age. On examination at 9 years of age, her height was on the 5th centile and weight on the 29th centile. The previously noted xanthomas had enlarged considerably to 2 to 4 cm. There were no signs of premature cardiovascular disease with normal coronary arteries on her echocardiogram, and her cardiovascular, respiratory and abdominal findings were unremarkable. In particular, there was no organomegaly or enlarged lymph nodes.

She was started on a low cholesterol and plant sterols diet. Ezetimide at 10 mg daily was initiated after 6 weeks of dietary therapy due to poor response (Table 1). Subsequently, cholesterol, sitosterol, and LDL‐cholesterol (LDL‐C) levels generally declined. There was a significant increase in total cholesterol after 6 months (2.8‐6.1 mmol/L). Based on dietary review, the patient had an intake of certain food(s) with higher fat content during that time. We believe that compliance with Ezetimibe was good but that these dietary factors, as well as the possibility of the high level being a nonfasting sample, may explain the increases seen.

## 3 DISCUSSION

Sitosterolemia is a rare and most likely underdiagnosed autosomal recessive disorder of lipid metabolism characterized by increased intestinal absorption and decreased biliary excretion of plant sterols due to variants in genes encoding the ATP‐binding cassette, subfamily G5 (*ABCG5*) or G8 (*ABCG8*).^2^ Digenic inheritance has also been described in an individual with heterozygous mutations in *ABCG5* and *ABCG8*.^12^ The clinical features are heterogeneous and the age of presentation varies between patients, making early diagnosis challenging.^4^ However, similar to our patient, dermatologic manifestation such as xanthomas may be the only presenting feature of sitosterolemia, and clinical suspicion must be high in order to recognize the condition.^13^ Patients with sitosterolemia are often diagnosed as a consequence of investigations for hypercholesterolemia. It should be noted that routine enzymatic cholesterol assays are also responsive to phytosterols. While cholesterol is increased in patients with sitosterolemia, the enzymatic measurement of cholesterol is an overestimation due to the significant contribution from phytosterols.

In our case, the correct diagnosis was delayed for more than 2 years after initial presentation and she was treated for familial hypercholesterolemia with poor response for a year. The patient was referred to Clinical Genetics for consideration for genetic testing of familial hypercholesterolemia to see if she would qualify for a PCSK9 inhibitor. The final diagnosis was made by targeted whole exome sequencing, highlighting the benefits of genomic medicine in rare clinical presentations.

The mechanism of action of the statins is primarily via inhibition of hydroxymethylglutaryl‐coenzyme A reductase, the rate‐limiting enzyme in the cholesterol biosynthesis pathway.^14^, ^15^ Our patient did not respond to statin therapy as expected. Her lipid profile was persistently abnormal.

Although no specific adverse symptoms emerged, this individual unfortunately did have a delay in the implementation of correct therapy, and therefore a prolonged duration of hyperlipidemia which could have caused adverse clinical consequences. This case illustrates the importance of clinicians reconsidering a presumed diagnosis in the face of poor treatment response.

Parents of the proband both demonstrated hypercholesterolemia, and both had positive family histories for hypercholesterolemia. Carriers of sitosterolemia typically have normal total cholesterol levels.^12^ The proband did not have any significant variants in known familial hypercholesterolemia (FH) genes (*APOB*, *LDLR*, *LDLRAP1*, *PCSK9*). It remains possible that one or other parent may carry a variant in one of these genes that was not passed on to the proband, since parents were only tested for the two *ABCG8* variants identified in their daughter. However, we consider it more likely that polygenic and lifestyle factors may be influencing the parental lipid levels.

The mainstay of therapy for sitosterolemia is dietary restriction of both cholesterol and plant sterols (vegetable oils, chocolate, margarine, avocado, nuts and seeds) and the use of ezetimibe (sterol absorption inhibitor).^16^ However, dietary restriction of plant sterols is very difficult to adhere to for most patients. Furthermore, growth retardation due to strict diet therapy may be a negative side effect in pediatric patients.^17^

The patient was tried on a low plant sterol and cholesterol diet for 6 weeks with insignificant response in lipid profile and therefore ezetimibe at 10 mg daily was added. Ezetimibe targets intestinal NPC1L1 transporter to inhibit absorption of sterols. It reduces plasma plant sterol levels and corrects most of the hematological abnormalities.^8^, ^18^ However, our patient did not have any hematological abnormalities.

The cutaneous lesions have remained clinically stable in size. Follow‐up imaging has not been obtained; however, we expect these lesions to decrease in size with time.^19^

This case illustrates the importance of considering rare conditions, such as sitosterolemia, as a differential diagnosis in patients with hypercholesterolemia and increased LDL‐C. Furthermore, when treatment is not producing the anticipated response, as in this case when simvastatin therapy did not improve the lipid profile, it is imperative that the presumptive diagnosis is reconsidered. It also highlights the clinical benefits of genomic sequencing in achieving an accurate diagnosis for unusual clinical presentations. Although various barriers exist to accessing genomic testing, limited exome sequencing is a cost‐effective and important tool in allowing accurate diagnosis and appropriate medical intervention for pediatric cases presenting with hypercholesterolemia in whom the differential diagnosis list is relatively short. Early targeted molecular genetic testing for individuals with rare and unusual clinical presentations allows accurate diagnosis and prognosis, initiation of appropriate therapy, reduction in unnecessary investigations leading to health economic benefits and also has the potential to reduce family anxiety and facilitate accurate genetic counseling around recurrence risks.

## CONFLICT OF INTEREST

The authors declare no conflicts of interest.

## AUTHOR CONTRIBUTIONS

Sharmila Kiss, Duncan MacGregor, Jane Wallace, Melanie Marty: Substantial contributions to the drafting, conception and design of the work, the acquisition, analysis, and interpretation of data for the work. Joy Yaplito Lee, Natasha J. Brown: Substantial contributions to the conception, design, and revision of the work critically for important intellectual content. Sharmila Kiss, Joy Yaplito Lee, James Pitt, Duncan MacGregor, Jane Wallace, Melanie Marty, Natasha J. Brown: Approved the version to be published and agree to be accountable for all aspects of the work in ensuring that questions related to the accuracy or integrity of any part of the work are appropriately investigated and resolved.

## ETHICS STATEMENT

Ethics committee approval not applicable.

## INFORMED CONSENT

All procedures were followed in accordance with the ethical standards of the responsible committee on human experimentations (institutional and national) and with the Helsinki Declaration of 1975, as revised in 2000. Informed and written consent for publication was obtained from the parents.

## ANIMAL RIGHTS

This article does not contain any studies with human or animal subjects performed by any of the authors.

## GUARANTOR OF THE ARTICLE

Natasha J. Brown.
